# Prevalence and Clinico-Pathologic Profiles of Nonodontogenic Cysts of the Oral and Maxillofacial Region: A Multicentre Study

**DOI:** 10.1155/ijod/4344848

**Published:** 2025-11-26

**Authors:** Soranun Chantarangsu, Promphakkon Kulthanaamondhita, Kittipong Dhanuthai, Kraisorn Sappayatosok, Poramaporn Klanrit, Nutchapon Chamusri, Pouyan Aminishakib, Seyed Mohammdmoein Hosseini, Ripon Singh, Jannat Uddin, Mark Darling

**Affiliations:** ^1^Department of Oral Pathology, Faculty of Dentistry, Chulalongkorn University, Bangkok, Thailand; ^2^Department of Oral Diagnostic Sciences, College of Dental Medicine, Rangsit University, Pathum Thani, Thailand; ^3^Department of Oral Biomedical Science-Division of Oral Diagnosis, Faculty of Dentistry, Khon Kaen University, Khon Kaen, Thailand; ^4^Department of Oral Biology and Oral Diagnostic Sciences, Faculty of Dentistry, Chiang Mai University, Chiang Mai, Thailand; ^5^Oral and Maxillofacial Pathology Department, School of Dentistry, Tehran University of Medical Sciences, Tehran, Iran; ^6^Department of Biology, Schulich School of Medicine and Dentistry, University of Western Ontario, Ontario, Canada; ^7^Department of Pathology and Laboratory Medicine, Schulich School of Medicine and Dentistry, University of Western Ontario, Ontario, Canada

**Keywords:** demographic, nonodontogenic cysts, pathologic profile, prevalence

## Abstract

**Objectives:**

To determine the prevalences, demographic, and pathologic profiles of patients diagnosed with nonodontogenic cysts (NOCs) in the oral and maxillofacial regions.

**Materials and Methods:**

Biopsy records from the participating institutions from 2000 to 2024 were studied for lesions diagnosed in the NOC category. Demographic profiles, the locations, and pathologic diagnoses were collected. Data were analyzed by using IBM SPSS Statistics version 29.0.

**Results:**

A total of 183,132 cases were obtained and 1864 cases (1.02%) were diagnosed as NOCs. The age of the patient ranged from 1 to 96 years with mean ± SD = 49.03 ± 18.43 years. The overall male-to-female ratio was 1.08:1. The majority of the lesions were encountered in the soft tissue. The most prevalent NOC was nasopalatine duct cyst followed by mucus retention cysts and nasolabial cysts.

**Conclusions:**

This study is the largest study on NOCs from Southeast Asia, the Middle East, and North America. The frequency of NOCs found in this studied population is somewhat different from those reported in previous studies. This study offers a valuable database for clinicians to facilitate the clinical differential diagnoses along with for the pathologists in rendering the final diagnosis.

## 1. Introduction

A cyst is a pathologic cavity that is lined by epithelium [1]. Numerous cysts of the head and neck region have been described. Within the oral and maxillofacial region, cysts are broadly classified into odontogenic cysts (OCs) and nonodontogenic cysts (NOCs) [2]. OCs originate from epithelium associated with tooth-forming apparatus or the entrapped remnants of this epithelium within bone or gingival tissue: the epithelial rests of Malassez or cell rests of Serres, respectively, whereas NOCs arise from ectodermal tissue involved in the development of orofacial tissue [2–4].

NOCs of the head and neck region are relatively uncommon and arise much less frequently than the OCs [4–6]. They are usually developmental in origin and comprise palatal cyst of the newborn, nasolabial cyst nasopalatine duct cyst, thyroglossal duct cyst, teratoid cyst, epidermoid and dermoid cysts, and oral lymphoepithelial cyst. However, certain NOCs may arise secondary to pathological conditions, for example, the mucus retention cyst of the salivary gland [1]. Some NOCs have historically been classified as fissural cysts based on the theory that they arise from the epithelium entrapped along embryonal lines of fusion [1]. However, this concept remains a subject of debate, and the precise histogenesis of several NOCs remains unresolved [7]. Once these cysts develop in the oral and maxillofacial region, their size tends to increase slowly, probably in response to a gradually elevated hydrostatic luminal pressure, and inflict minimal damage to the adjacent structures [1]. Some lesions may resolve spontaneously, whereas others may have a propensity to recur if not treated adequately.

The classification of NOCs is not absolute and is constantly changing. Its classification has been changed over the years with some lesions being obsolete due to inappropriate diagnosis and no longer exist [1]. The globulomaxillary cyst, previously portrayed as a fissural cyst caused by the entrapment of epithelium between the nasal and maxillary processes, is no longer considered as an entity [8]. Median mandibular cyst no longer exists as an entity since previous reports may be the consequence of misdiagnosis of other OCs [9]. The 2017 WHO Classification of Head and Neck Tumours only included one NOC in the “odontogenic and non-odontogenic developmental cysts” section: the nasopalatine duct cyst [10]. The most recent WHO Classification of Head and Neck Tumours in 2022 has changed the title of this section into “Cysts of the Jaw.” However, the entity of NOC, the nasopalatine duct cyst, included in this section remained unchanged [11].

Previous studies have demonstrated different demographics and characteristics of the NOCs across various populations [4–6, 12–14]. In the Brazilian population, the most commonly encountered NOCs were nasopalatine duct cysts, epidermoid cysts and oral lymphoepithelial cysts, respectively, and were predominantly found in females [5]. As for the Turkish population, among the 5088 cases studied, the majority of the cases were OCs with only 1.7% of the cases were diagnosed as NOCs [4]. Another study in the Brazilian population by Franklin et al. [13] also showed similar results with the prevalence of 7.1% with epidermoid cyst as the most prevalent NOC, followed by oral lymphoepithelial cyst and nasopalatine duct cyst, in concurrence with another study in children and adolescents of a Brazilian population [12]. One study in the Thai population revealed different prevalence of NOCs, showing mucocele as the most common lesion followed by nasopalatine duct cyst [14]. From all these findings, it seems that the prevalence of the NOCs was different across populations and the differences in diagnosis criteria of the NOCs also play an important part.

Our group previously published a multicentre study about the cysts of the jaw, which included only two NOCs that located within the jawbone [15]. However, there are other NOCs that located outside the jaw but within the oral and maxillofacial region that have not been investigated. As mentioned, the prevalence of NOCs is generally low. Owing to a small number of studies on NOCs in the literature, the information regarding the demographic profile of these lesions in different populations, particularly in Southeast Asia, is limited. Therefore, the aim of this study is to perform a comprehensive analysis of NOCs from the participating institutions in Southeast Asia, the Middle East, and North America and to compare our findings with published data worldwide.

## 2. Materials and Methods

The study was approved by the Human Research Ethics Committee of Rangsit University (RSUERB2025-007). Biopsy records were obtained from the Department of Oral Pathology, Chulalongkorn University; Department of Oral Biology and Diagnostic Sciences, Chiang Mai University; Department of Oral Diagnosis, Khon Kaen University; Department of Oral and Maxillofacial Pathology, Tehran University of Medical Sciences and Department of Pathology and Laboratory Medicine, University of Western Ontario, and Department of Oral Diagnostic Sciences, Rangsit University. Cystic lesions that were diagnosed during 2000–2024 were reviewed from the biopsy records and demographic data were extracted.

For age of the participants, individuals with the age of 16 and below were classified as pediatric participants, whereas those aged 65 and above were classified as geriatric participants.

For location of lesions, two regions of the jaw were used: maxilla and mandible. Respective anatomical sites of soft tissue specimens retrieved from the oral and maxillofacial were recorded, including, floor of the mouth, tongue, lip, palate, buccal mucosa, maxillary sinus, nasolabial area, orofacial skin, and tonsil. Lastly, the lesions from sites that were not listed above were categorized as “other location.”

All cysts were evaluated and diagnosed according to the 5th edition of Oral and Maxillofacial Pathology [1]. Every case included in this study was carefully reviewed by board certified oral pathologists. The histopathologic criteria for each disease entity were set to standardized the diagnostic criteria across centers (Table S1). Incisional biopsies and surgical specimens along with the recurrent lesions from the same patient with the same diagnosis were combined and considered as one diagnosis for this study.

### 2.1. Statistical Analysis

The descriptive statistics of means and standard deviations were used for a numerical variable (age) whereas for categorical variables (sex and location), frequencies, and percentages were employed. To account for the sampling imbalance among regions, the weighted prevalence for each diagnosis was calculated by an inverse variance random-effects model using the meta package in R Statistics version 4.5.1 (R Foundation). Welch one-way analysis of variance (ANOVA) followed by Games-Howell post-hoc analysis was used for the differences in mean age among lesions. One sample chi-square test was used to analyze the differences in the sex proportion within each lesion. IBM SPSS Statistics version 29.0 (IBM) was utilized to perform all the analysis except calculating the weighted prevalences. A statistically significant difference was determined with a *p*-value of 0.05.

## 3. Results

Of the total of 183,132 accessioned cases, 1864 cases (1.02%) were diagnosed in the group of NOCs. The age of the study population ranged from 1 to 96 years with mean ± SD = 49.03 ± 18.43 years. The majority of the participants (68.05%) were in the 4th to the 7th decades of life. Pediatric patients (≤16 years) accounted for 4.40% of all the cases, while geriatric patients (≥65 years) comprised 21.51%. Of the total, 968 (51.96%) were male and 895 (48.04%) were female, with an overall male-to-female ratio of 1.08:1. Males slightly outnumbered females in both the pediatric (1.22:1) and geriatric (1.07:1) subgroups. Overall, NOCs were most commonly located in soft tissue (56.2%). According to the anatomic sites, the NOCs were most frequently found in the maxilla (39.80%), followed by the floor of the mouth (12.90%) and the tongue (11.90%), respectively.

Table 1 presents the distribution of NOCs overall and by country. A total of 1864 NOC cases were analyzed from three different countries: Canada (*n* = 1492), Iran (*n* = 113), and Thailand (*n* = 259). The prevalence of NOCs was highest in Iran (1.87%), followed by Canada (1.08%), and lowest in Thailand (0.67%). According to the weighted prevalence, the most common type of NOCs across all regions was nasopalatine duct cyst (48.01%), followed by mucus retention cysts (16.79%) and nasolabial cysts (10.48%), respectively. Nasopalatine duct cyst was the most frequently diagnosed cyst in all three countries, accounting for 58.30% of cases in Thailand, 53.10% in Iran, and 33.65% in Canada. Mucus retention cysts were more prevalent in Canada (30.90%) compared to Iran (10.62%) and Thailand (8.49%). In contrast, nasolabial cysts were relatively rare in Canada (0.80%), but showed a higher occurrence in Thailand (17.37%) and Iran (14.16%).

Table 2 reveals the distribution of age and sex according to the pathological diagnosis of NOCs. Among individual cyst types, nasolabial cysts had the highest mean age (56.62 ± 16.38 years), followed by dermoid cysts (55.38 ± 18.28 years) and mucus retention cysts (51.94 ± 20.27 years). In contrast, sinus retention cysts demonstrated the lowest mean age (36.18 ± 18.78 years), followed by sebaceous cysts (36.80 ± 27.09 years) and trichilemmal cysts (39.00 ± 12.73 years). Nasolabial cysts were associated with a significantly older age compared to nasopalatine duct cysts, oral lymphoepithelial cysts, epidermoid cysts, surgical ciliated cysts, and sinus retention cysts (*p*=0.008, 0.003, <0.001, 0.019, and 0.002, respectively). Additionally, sinus retention cysts were associated with a significantly younger age compared to mucus retention cysts (*p*=0.023). Among the pediatric participants, mucus retention cysts (39.02%) represented the most prevalent pediatric NOCs, followed by nasopalatine duct cysts (28.05%) and oral lymphoepithelial cysts (21.95%). Similarly, in the geriatric participants, mucus retention cysts (37.91%) were the most frequent geriatric NOCs, followed by nasopalatine duct cysts (34.16%) and oral lymphoepithelial cysts (13.47%). A male predominance was observed in nasopalatine duct cysts (M:F = 1.69:1) and epidermoid cysts (M:F = 1.44:1), both of which showed significant differences in sex distribution (*p* < 0.001 and 0.025, respectively). In contrast, oral lymphoepithelial cysts (M:F = 0.65:1) and nasolabial cysts (M:F = 0.28:1) were more common in females (*p* < 0.001). Other cysts showed no significant sex differences.

Table 3 reports the distribution site of NOCs. Nasopalatine duct cysts were exclusively found in the anterior maxilla, whereas mucus retention cysts had a wider distribution, with the highest occurrence in the floor of the mouth (27.07%), followed by the lip (20.61%) and palate (18.18%). Oral lymphoepithelial cysts were most commonly located on the tongue (55.87%) and floor of the mouth (25.50%). Epidermoid cysts were frequently found on the orofacial skin (30.77%) and the lip (33.97%). Nasolabial cysts were exclusively present in the nasolabial area (100%), while surgical ciliated cysts were predominantly found in the maxillary sinus (84.62%).

## 4. Discussion

This study represents the largest series of NOCs encompassing both the intraosseous and soft tissue cysts in the oral and maxillofacial region from Southeast Asia, the Middle East, and North America. Previous studies have demonstrated higher prevalence of the OCs [16, 17] with a small number of NOCs, particularly the nasopalatine duct cyst [5, 6, 15]. However, there are other NOCs that should be accounted for, since presentation could mimic odontogenic lesions and in an appropriate situation, should be included in the differential diagnosis. Therefore, knowledge of the variability of NOCs is crucial and important to be acknowledged. Nevertheless, it is somewhat difficult to compare the findings of the current study with those of the previous studies due to the different classifications and inclusion criteria used.

The results of our study showed that NOCs constituted 1.02% of all the accessioned cases. This is in accordance with 0.1%−0.56% in previous studies [4, 5, 12, 13], but lower than 6% reported by Kriangcherdsak et al. [14]. The potential impact of underdiagnosis should be taken into consideration since some of the asymptomatic NOC cases are not submitted for histopathological diagnosis. This could lead to an underestimation of the true prevalence. The most frequent NOC in our study was nasopalatine duct cyst which constituted to 38.25% of all accessioned cases. This is consistent with previous study by Nonaka et al. [5], which reported the prevalence of 32.8%, but higher than 17.76% reported by Kriangcherdsak et al. [14]. However, when compared with other studies, our findings show a much higher percentage of nasopalatine duct cyst [12, 13]. The second most common NOC in our study was mucus retention cyst, which constituted 26.56% of all NOCs and is much higher than the previous study by Kriangcherdsak et al. [14]. Lastly, oral lymphoepithelial cyst constituted 18.72% of all NOCs, similar to previous studies [5, 13] but higher than 0.9% reported by da Silva et al. [12]. A plausible reason for the variability in the frequencies and percentages when compared to previous studies could be due to the method of comparison, where the number of NOC was compared to the total number of lesions, whereas in our study the comparison was made only among the NOCs. In addition, the demographics and characteristics of the NOCs across various populations could also contribute to these findings.

The average age of the patients with NOCs in this study was 49.03 years, higher than all previous studies, which ranged from 32 to 39 years [5, 12–14]. Regarding the most common NOC from our study, nasopalatine duct cyst, the mean age was 56.62 years; also, higher than all previous studies, which reported the mean age between 46.2 and 52 years [5, 12–14]. The mean age of the patients with mucus retention cyst was 51.94 years which is lower than 61 years reported by Kriangcherdsak et al. [14] Lastly, the average age of the patients with oral lymphoepithelial cysts was 47.70 years which is comparable to 45.57 years reported by Frankin et al. [13] but higher than 15.0 and 39.8 years reported by da Silva et al. [12] and Nonaka et al. [5], respectively.

Overall, the NOCs were equally distributed among males and females, which is in accordance with previous studies [12–14]. However, a male predominance was also observed in nasopalatine duct cysts, which is in agreement with previous studies [12–14]. Contrary to this study, Nonaka et al. [5] found that NOCs were mainly observed in females with M:F ratio of 1:1.9 as well as the predominance of nasopalatine duct cyst in females (M:F = 1:2). Oral lymphoepithelial cysts were encountered in females more than in males, in accordance with all previous reports [5, 12–14].

With regard to the anatomical distribution of NOCs, the majority of the lesions were encountered in the soft tissue with floor of the mouth as the most common site followed by tongue and lips, respectively. For intraosseous NOCs, the maxilla is affected more than the mandible. Nasopalatine duct cyst was exclusively found in the anterior maxilla and is in total agreement with previous studies [12–14]. Mucus retention cyst was most commonly found in floor of the mouth in this study, while Kriangcherdsak et al. [14], reported that mucus retention cysts were equally observed in the buccal mucosa and palate. Our findings demonstrated that oral lymphoepithelial cyst was mostly encountered at the tongue similar to a study by da Silva et al. [12] but different from the study by Franklin et al. [13], which reported higher frequency in the floor of the mouth and palate.

As mentioned, the prevalence of some lesions in this study differed from previous reports. This could be due to the differences in patient demographics and population, as well as the classification of NOCs. Clinical presentations, radiographic features coupled with knowledge of the prevalence, site of predilection, age, and gender distribution may aid clinicians in formulating the most probable clinical diagnosis. Nevertheless, the final diagnosis relies on the microscopic examination of the biopsied specimen. All surgically removed tissues should be submitted for histopathological examination to ensure an accurate final diagnosis. However, in some cases, diagnosis of NOCs can be challenging due to their quiescent development and the similarity in clinical and radiographic features to other lesions. The asymptomatic nature of these cysts could delay the diagnosis and treatment. However, the progressive growth of these cysts could lead to the destruction of adjacent vital structures and cause adverse esthetic issues and functional defects to the orofacial structures. Therefore, to avoid these complications, an accurate diagnosis and appropriate treatment modalities are necessary. Knowledge of the clinical characteristics, histopathologic features, and behaviors of cystic lesions is the key for ensuring an early and accurate clinical diagnosis, which will lead to prompt treatment and a favorable long-term prognosis and outcome.

In conclusion, this study is the largest study on NOCs from Southeast Asia, the Middle East, and North America. The frequency of NOCs found in this studied population is somewhat different from those reported in previous studies. Nasopalatine duct cysts, mucus retention cysts and oral lymphoepithelial cysts were the most common NOCs found, accounting for over 80%. This study offers a valuable database for clinicians to facilitate the clinical differential diagnoses along with for the pathologists in rendering the final diagnosis.

## Figures and Tables

**Table 1:** Frequencies and percentages of nonodontogenic cysts according to country regions.

Diagnosis	Canada	Iran	Thailand	Total	
	*N* (%)	*N* (%)	*N* (%)	*N* (%)	% Weighted prevalence (95% confidence interval)^a^
Nasopalatine duct cyst	502 (33.65)	60 (53.10)	151 (58.30)	713 (38.25)	48.01 (32.84–63.19)
Mucus retention cyst	461 (30.90)	12 (10.62)	22 (8.49)	495 (26.56)	16.79 (2.64–30.93)
Nasolabial cyst	12 (0.80)	16 (14.16)	45 (17.37)	73 (3.92)	10.48 (0.18–20.78)
Oral lymphoepithelial cyst	342 (22.92)	4 (3.54)	3 (1.16)	349 (18.72)	9.22 (0–22.76)
Epidermoid cyst	117 (7.84)	15 (13.27)	24 (9.27)	156 (8.37)	8.71 (6.67–10.75)
Surgical ciliated cyst	20 (1.34)	6 (5.31)	13 (5.02)	39 (2.09)	3.44 (0.63–6.26)
Sinus retention cyst	22 (1.47)	0 (0)	0 (0)	22 (1.18)	0.54 (0–1.55)
Dermoid cyst	8 (0.54)	0 (0)	0 (0)	8 (0.43)	0.27 (0–0.75)
Sebaceous cyst	4 (0.27)	0 (0)	1 (0.39)	5 (0.27)	0.27 (0.03–0.51)
Tricholemmal cyst	2 (0.13)	0 (0)	0 (0)	2 (0.11)	0.12 (0–0.29)
Branchial cleft cyst	1 (0.07)	0 (0)	0 (0)	1 (0.05)	0.06 (0–0.19)
Thyroglossal duct cyst	1 (0.07)	0 (0)	0 (0)	1 (0.05)	0.06 (0–0.19)
Total	1492 (100)	113 (100)	259 (100)	1864 (100)	—

^a^Calculated by an inverse variance random-effects model using the meta package in R Statistics version 4.5.1.

**Table 2:** Basic characteristics including age and sex distribution according to the pathological diagnosis of nonodontogenic cysts from the highest to the lowest frequencies.

Diagnosis	Age (year)		Sex			
	Mean ± SD	Range	Male *N* (%)	Female *N* (%)	M:F ratio	*p*-Value*⁣*^*∗*^
Nasopalatine duct cyst	48.69 ± 17.34	1–96	448 (62.83)	265 (37.17)	1.69:1	**<0.001**
Mucus retention cyst	51.94 ± 20.27	5–88	234 (47.37)	260 (52.63)	0.90:1	0.242
Nasolabial cyst	56.62 ± 16.38	23–94	16 (21.92)	57 (78.08)	0.28:1	**<0.001**
Oral lymphoepithelial cyst	47.70 ± 17.29	4–86	138 (39.54)	211 (60.46)	0.65:1	**<0.001**
Epidermoid cyst	43.87 ± 18.28	4–95	92 (58.97)	64 (41.03)	1.44:1	**0.025**
Surgical ciliated cyst	45.62 ± 14.71	25–79	19 (48.72)	20 (51.28)	0.95:1	0.873
Sinus retention cyst	36.18 ± 18.78	11–73	11 (50)	11 (50)	1:1	1.000
Dermoid cyst	55.38 ± 18.28	18–80	5 (62.5)	3 (37.50)	1.67:1	0.480
Sebaceous cyst	36.80 ± 27.09	12–79	2 (40)	3 (60)	0.67:1	0.655
Tricholemmal cyst	39.00 ± 12.73	30–48	1 (50)	1 (50)	1:1	1.000
Branchial cleft cyst	20	N/A	1 (100)	0 (0)	N/A	N/A
Thyroglossal duct cyst	33	N/A	1 (100)	0 (0)	N/A	N/A
Total	49.03 ± 18.43	1–96	968 (51.96)	895 (48.04)	1.08:1	0.091

*⁣* ^*∗*^Difference of proportions between male and female using one-sample chi-square test. Statistically significance difference indicates by bold (*p* < 0.05).

Abbreviation: N/A, not available.

**Table 3:** Distribution of nonodontogenic cysts according to anatomic sites.

Diagnosis	Maxilla	Mandible	Floor of mouth	Tongue	Lip	Palate	Buccal mucosa	Maxillary sinus	Nasolabial area	Orofacial skin	Tonsil	Other
Nasopalatine duct cyst	713	0	0	0	0	0	0	0	0	0	0	0
Mucus retention cyst	18	40	134	22	102	90	77	0	0	0	1	11
Nasolabial cyst	0	0	0	0	0	0	0	0	73	0	0	0
Oral lymphoepithelial cyst	1	2	89	195	3	32	0	0	0	0	20	7
Epidermoid cyst	4	5	16	3	53	6	16	0	0	48	0	5
Surgical ciliated cyst	6	0	0	0	0	0	0	33	0	0	0	0
Sinus retention cyst	0	0	0	0	0	0	0	22	0	0	0	0
Dermoid cyst	0	1	1	1	4	0	0	0	0	1	0	0
Sebaceous cyst	0	0	0	0	1	0	1	0	0	1	0	2
Tricholemmal cyst	0	0	0	0	0	0	0	0	0	1	0	1
Branchial cleft cyst	0	0	0	0	0	0	0	0	0	1	0	0
Thyroglossal duct cyst	0	0	1	0	0	0	0	0	0	0	0	0
Total *N* (%)	742 (39.80)	48 (2.60)	241 (12.90)	221 (11.90)	163 (8.70)	128 (6.90)	94 (5.00)	55 (3.00)	73 (3.90)	52 (2.80)	21 (1.10)	26 (1.40)

## Data Availability

The data that support the findings of this study are available from the corresponding author upon reasonable request.

## Supporting Information

Supporting Information Table S1. Describes the histopathologic criteria for diagnosis of nonodontogenic cysts in this study. Table S2. Demonstrates the distribution of age groups and sex according to diagnosis of nonodontogenic cysts.
