# Supplementary material for: Prevalence and Clinico-Pathologic Profiles of Nonodontogenic Cysts of the Oral and Maxillofacial Region: A Multicentre Study
Source: Int J Dent. 2025 Nov 26;2025:4344848. doi: 10.1155/ijod/4344848 (PMC12674867; doi:10.1155/ijod/4344848)
Supplement: Supporting Information — Table S1. Describes the histopathologic criteria for diagnosis of nonodontogenic cysts in this study. Table S2. Demonstrates the distribution of age groups and sex according to diagnosis of nonodontogenic cysts. [file 4344848.f1.docx]

**Supplementary Materials**

**Prevalence and clinico-pathologic profiles of non-odontogenic cysts of the oral and maxillofacial region: A multicentre study**

**Supplementary Table 1** Histopathologic criteria for diagnosis of non-odontogenic cysts

| **Diagnosis** | **Histopathologic criteria** |
| --- | --- |
| Nasopalatine duct cyst | - Located at anterior maxilla - Epithelial lining composed of   - Stratified squamous epithelium and/or   - Pseudostratified columnar epithelium and/or   - Simple columnar epithelium and/or   - Simple cuboidal epithelium - Moderate-sized nerves and small muscular arteries and veins in the cyst wall |
| Mucus retention cyst | - Cuboidal, columnar, or atrophic squamous epithelial lining - Thin or mucoid secretions in the lumen |
| Oral lymphoepithelial cyst | - Cystic cavity that is lined by stratified squamous epithelium without rete ridges - Presence of lymphoid tissue in the cyst wall |
| Epidermoid cyst | - Lined by stratified squamous epithelium with well-developed granular cell layer - The lumen is filled with degenerating orthokeratin |
| Nasolabial cyst | - Located in soft tissue - Lined by pseudostratified columnar epithelium with/without goblet cells and cilia |
| Surgical ciliated cyst | - History of trauma or previous surgery in this area - Lined by ciliated pseudostratified columnar epithelium, squamous epithelium with mucous cells, or metaplastic squamous epithelium |
| Sinus retention cyst | - Focal dilation of a duct associated with the seromucous glands of the sinus lining - The lumen of the dilated duct is filled with thick mucus and chronic inflammatory cells |
| Dermoid cyst | - Lined by orthokeratinized stratified squamous epithelium with a prominent granular cell layer - Abundant keratin within the cyst lumen - The cyst wall is composed of fibrous connective tissue that contains one or more skin appendages |
| Sebaceous cyst | - Lined by orthokeratinized stratified squamous epithelium with a prominent granular cell layer - Abundant keratin within the cyst lumen |
| Tricholemmal cyst | - lined by stratified squamous epithelium - The keratinocytes remain large in the upper epithelial layers with an abrupt transition to dense, compact keratin that fills the cyst lumen |
| Branchial cleft cyst | - Lined by stratified squamous epithelium - The wall of the cyst contains lymphoid tissue with germinal center formation |
| Thyroglossal duct cyst | - Lined by respiratory or stratified squamous epithelium - Thyroid tissue in the cyst wall |

**Supplementary Table 2** Distribution of age groups and sex according to diagnosis of non-odontogenic cysts

| **Diagnosis** | **Age groups** | **Male, n (%)** | **Female, n (%)** | ***p*-value*** |
| --- | --- | --- | --- | --- |
| Nasopalatine duct cyst | ≤ 16 | 10 (2.2) | 13 (4.9) | 0.137 |
|  | 17 - 64 | 349 (77.9) | 204 (77.0) |  |
|  | ≥ 65 | 89 (19.9) | 48 (18.1) |  |
| Mucus retention cyst | ≤ 16 | 22 (9.4) | 10 (3.8) | **0.039** |
|  | 17 - 64 | 145 (62.0) | 166 (63.8) |  |
|  | ≥ 65 | 67 (28.6) | 84 (32.4) |  |
| Nasolabial cyst | ≤ 16 | 0 (0) | 0 (0) | 0.365 |
|  | 17 - 64 | 9 (56.2) | 39 (68.4) |  |
|  | ≥ 65 | 7 (43.8) | 18 (31.6) |  |
| Oral lymphoepithelial cyst | ≤ 16 | 9 (6.5) | 9 (4.3) | 0.126 |
|  | 17 - 64 | 102 (73.9) | 175 (82.9) |  |
|  | ≥ 65 | 27 (19.6) | 27 (12.8) |  |
| Epidermoid cyst | ≤ 16 | 2 (2.1) | 4 (6.2) | 0.186 |
|  | 17 - 64 | 79 (85.9) | 48 (75.0) |  |
|  | ≥ 65 | 11 (12.0) | 12 (18.8) |  |
| Surgical ciliated cyst | ≤ 16 | 0 (0) | 0 (0) | 0.267 |
|  | 17 - 64 | 16 (84.2) | 19 (95.0) |  |
|  | ≥ 65 | 3 (15.8) | 1 (5.0) |  |
| Sinus retention cyst | ≤ 16 | 1 (9.1) | 1 (9.1) | 1.000 |
|  | 17 - 64 | 9 (81.8) | 9 (81.8) |  |
|  | ≥ 65 | 1 (9.1) | 1 (9.1) |  |
| Dermoid cyst | ≤ 16 | 0 (0) | 0 (0) | 0.187 |
|  | 17 - 64 | 4 (80.0) | 1 (33.3) |  |
|  | ≥ 65 | 1 (20.0) | 2 (66.7) |  |
| Sebaceous cyst | ≤ 16 | 1 (50.0 | 0 (0) | 0.082 |
|  | 17 - 64 | 0 (0) | 3 (100) |  |
|  | ≥ 65 | 1 (50.0) | 0 (0) |  |
| Tricholemmal cyst | ≤ 16 | 0 (0) | 0 (0) | N/A |
|  | 17 - 64 | 1 (100) | 1 (100) |  |
|  | ≥ 65 | 0 (0) | 0 (0) |  |
| Branchial cleft cyst | ≤ 16 | 0 (0) | 0 (0) | N/A |
|  | 17 - 64 | 1 (100) | 0 (0) |  |
|  | ≥ 65 | 0 (0) | 0 (0) |  |
| Thyroglossal duct cyst | ≤ 16 | 0 (0) | 0 (0) | N/A |
|  | 17 - 64 | 1 (100) | 0 (0) |  |
|  | ≥ 65 | 0 (0) | 0 (0) |  |

* Association between age groups and sex was analyzed using chi-square test. Statistically significance difference indicates by bold (*p* < 0.05).

Abbreviations : N/A, not available.
